# Hepatitis B Knowledge, Testing, and Vaccination History among Undergraduate Public Health Students in Ghana

**DOI:** 10.1155/2019/7645106

**Published:** 2019-08-14

**Authors:** Eric Osei, John Niyilapah, Gregory Kofi Amenuvegbe

**Affiliations:** ^1^Department of Population and Behavioural Sciences, School of Public Health, University of Health and Allied Sciences, Hohoe, Ghana; ^2^Department of Epidemiology, School of Public Health, University of Health and Allied Sciences, Hohoe, Ghana; ^3^Department of Health Policy, Planning and Management, School of Public Health, University of Health and Allied Sciences, Hohoe, Ghana

## Abstract

**Background:**

Hepatitis B virus (HBV) infection is a serious public health problem in many parts of the world. The risk of acquiring the infection through exposure to blood, semen, and other bodily fluids is highest among health care workers (HCW) including trainees. Ghana is considered a high risk country for HBV; however little is known about the knowledge and prevention practices of the infection in the country. This study assessed the knowledge, testing, and vaccination history of HBV and their related factors among undergraduate public health students of University of Health and Allied Sciences in Ghana.

**Methods:**

A cross-sectional study was conducted among 226 students using a pretested questionnaire to assess Hepatitis B knowledge, testing, and vaccination history of the students. We performed logistic regression analysis to examine the relationship between Hepatitis B testing and vaccination history and participants' characteristics. Data was analysed using Stata Version 12.

**Results:**

Majority 169 (73.9%) of the 226 participants studied had moderate knowledge regarding HBV infection. About half 114 (50.4%) of them had never been tested for HBV infection, and 100 (44.2%) had received at least a single dose of Hepatitis B vaccine. The completed vaccination rate among the students was 30.5%. Students in their 2nd year (Adjusted Odds Ratio [AOR]: 3.13; 95% Confidence Interval [CI]: 1.13, 7.52; p<0.011) and those with moderate (AOR: 4.76; 95% CI; 1.35, 16.82; P=0.015) and good (AOR: 5.40; 95% CI: 1.31, 22.36; P=0.020) level of knowledge were more likely to be tested for HBV. With regard to vaccination, females (AOR: 1.85; 95%CI: 1.04-3.29; P=0.037) and regular students (AOR: 0.37; 95%CI: 0.19, 0.70; p=0.002) were associated with receiving the full dose of Hepatitis B vaccine.

**Conclusion:**

This study highlights the urgent need for continued health education on HBV infection and strategies that ensure that health trainees are screened and fully vaccinated against the infection to prevent potential future exposure to the virus. The students' representative council can organize free HBV testing and vaccination for all fresh students.

## 1. Background

The hepatitis B virus (HBV) infection is highly contagious and is transmitted by percutaneous or mucosal exposure to infected blood, semen, or other bodily fluids. Infants born to untreated HBV-infected mothers can also acquire the infection from the mother during birth [1]. The burden of HBV constitutes a public health threat in many parts of the world, despite the availability of an effective vaccine and treatments [2]. According to the World Health Organization (WHO), there were an estimated 257 million people living with chronic HBV infection in 2015 globally, with about 887,000 of them dying from the disease. The burden of HBV infection is particularly higher in sub-Saharan Africa and Western Pacific regions. [3]

Ghana is classified as part of the areas of the world where the burden of HBV infection is considered to be high [4, 5]. Asenso-Ofori and colleagues, for instance, in their systematic review, estimated the prevalence of HBV infection among Ghanaian population to be 12.3% [6]. Similarly, Apama et al. classified Ghana as HBV highly endemic country with an estimated prevalence of 12.92%, a rate higher than that of countries like Cote d'lvoire, Togo, Nigeria, and Burkina Faso [3]. Osei et al. [7] also estimated the prevalence of the HBV among blood donors to be 7.5% in the Volta region. This suggests that HBV is still a significant public health problem in the country which requires greater attention.

HBV is an important occupational hazard for unvaccinated HCWs including students, with an estimated 2 to 4 times higher incidence than the general population [2, 7, 8]. Knowledge regarding the infection and its related safety precautions among HCWs is therefore essential to minimize the acquired infections in healthcare settings among this vulnerable population as they remain in direct contact with potentially infected persons, blood, and medical tools and instruments during the course of service delivery [9, 10]. Inadequate knowledge of HBV among HCWs, however, may affect their behavioural pattern to vaccination and safety measures.

In spite of the high burden of disease due to chronic Hepatitis B and the improvements and opportunities for care and treatment worldwide, majority of people infected with HBV are unaware of their status and hence normally present with advanced stage of the disease [2]. In the low-income countries, for instance, only about 5% of HBV-infected persons know their infection status. This low uptake of HBV testing is as a result of inadequate testing facilities or services, lack of effective policies and standards for testing, poor laboratory capacity and infrastructure, high cost and complex diagnostic assays and algorithms, and the use of substandard test kits and reagents [2].

The HBV vaccines (HBVc) have been in existence since 1992 and are available as monovalent formulations for birth doses or for vaccination of at risk adult populations [2, 11]. A standard three-dose vaccine regimen, with the second and third doses given 1 month and 6 months apart, respectively, from the initial dose, has been identified as very effective in conferring immunity against HBV [12, 13]. Among HCWs and other healthy adults, HBV vaccination is known to be highly effective for prevention of the infection; hence HBV vaccination has been recommended by the WHO as a primary preventive strategy for the control of the infection among HCWs [14–17].

Many studies have estimated HBV knowledge and vaccination status among HCWs and medical students in many parts of the world [18–22]. Little evidence is however available in Ghana. To bridge the knowledge gap, this study was conducted among undergraduate public health students of University of Health and Allied Sciences, Ghana.

## 2. Materials and Methods

### 2.1. Study Design and Setting

A cross-sectional study among undergraduate public health students of University of Health and Allied Sciences was conducted in February, 2017. The University is one of the public institutions in Ghana, established in 2012 to offer higher education to undergraduates and graduates in Health related fields including medicine, Nursing, Midwifery, Pharmacy and other Allied Health Sciences. The University currently has six schools/colleges including the School of Public Health, where this study was conducted. The School of Public Health is located in Hohoe and currently trains undergraduates and graduates in the field of Public Health. The school offers admission to qualified candidates from the Senior Secondary Schools (regular students) into any of the Bachelor of Public Health programs. The school also offers top-up degree programs for healthcare professionals with diploma or certificate in health related fields (top-up students). There are currently about 700 students enrolled in programs such as Disease Control, Health Promotion, Public Health Nutrition, Environmental Health, and Health Information. Master of Public health and Master of Philosophy in Allied Epidemiology are the two graduate courses offered by the school.

### 2.2. Sample Size and Sampling Procedure

The sample size used for this study was 226 students. This was based on the assumption of 56% of students with adequate knowledge about HBV [23], at 95% confidence level, and 5% margin of error using StatCal option of Epi Info Version 7 and adjusting for a nonresponse rate of 5%. Stratified sampling was used to select the study participants from different year/level of study and different track to ensure representativeness. The number of students selected from each year/level was proportional to the students' population.

### 2.3. Instrument and Data Collection

The study was carried out using a structured pretested questionnaire written in English. The questionnaire consisted of three parts: (i) demographic characteristics of the respondents; (ii) Hepatitis B testing and vaccination status; (iii) knowledge about Hepatitis B infection. The questionnaire assessed the respondents' general knowledge regarding hepatitis B virus infection, mode of transmission, signs and symptoms, treatment, and preventive measures. The history of HBV testing and vaccination was also assessed through self-reporting. The questionnaires were administered to participants during their free time on the school campus or in their hostels by trained National Service personnel after obtaining informed consent. To enhance data quality and accuracy, the questionnaire was pretested among 20 students in the same school who were excluded from the final study. All inconsistencies and wrong wordings in the questions identified were corrected before the final administration to study participants.

### 2.4. Data Analysis

The data was entered into Epi Data Version 3.1 for validation and cleaning. Analysis was carried out using Stata statistical package Version 12 (Stata Corp, Collage Station). Descriptive statistics were used to describe the study population in relation to relevant variables. Beyond descriptive statistics, both univariate and multivariate logistic regression models were used to identify factors associated with dependent variables (Hepatitis B testing and vaccination “status”) and independent variables. Odds ratio and 95% Confidence Intervals (95%CI) were computed. First, the potential predictors were evaluated for their individual association with the dependent variables in a univariate analysis. Secondly, multiple logistic regression analysis was performed to adjust for possible confounding effect of predictors associated with Hepatitis B testing and vaccination. All predictor variables that showed association with outcome variables (p < 0.05) in the univariate analysis were included in the multivariable model. Vaccination history was modelled as complete vaccination (receiving 3+ doses) and incomplete vaccination (receiving 0-2 doses). The composite measure of students' knowledge was measured by the total number of correct answers to 19 items on knowledge of Hepatitis B. Knowledge was measured using a scoring system where a value of one was assigned to each correct knowledge item and zero for a wrong knowledge item. Knowledge score of 16-19 was considered good knowledge, score of 10-15 was considered moderate knowledge, and a score of less than 10 was classified as having poor knowledge.

### 2.5. Ethical Issues

This study was ethically approved by the Ethical Review Committee of Ghana Health Service. Permission was sought from the University authorities before data collection. Written informed consent was obtained from each respondent after information about the study have been read and explained to them before interviews were conducted. Confidentiality and privacy were ensured.

## 3. Results

### 3.1. Characteristics of the Study Participants

Of the 226 students who participated in the study, 153 (67.7%) were males and the rest females. The ages of the participants ranged from 18 to 42 years with the mean (standard deviation (SD)) age of 24.12 ± 5.01 years. The majority of them 160 (70.8%) were between 20 and 29 years, 195 (86.3%) were never married, and 174 (76.9%) were regular students, while the rest were top-up students, and 181(80.1%) permanently resided in urban areas (Table 1).

### 3.2. Knowledge on HBV Transmission Dynamics

The mean (SD) knowledge score was 12.98 ± 2.72. Overall, 39 (17.3%) of the students had good knowledge regarding HBV infection. About three-quarters 169 (73.9%) and 20 (8.9%) of them had moderate and poor knowledge, respectively.

With regard to participants' knowledge on HBV transmission dynamics, 145 (65.9%) of them correctly said HBV can be transmitted through unprotected sexual intercourse, 183 (83.2%) knew blood transfusion, and 138 (62.7%) affirmed that people can get HBV infection by sharing towel with an infected person. More than half of the students 125 (56.7%) knew that HBV cannot be transmitted through faeco-oral route and 167 (75.9%) knew that people can acquire the infection by sharing sharps with an infected person, while 171 (53.2%) said HBV is not hereditary. In addition, 140 (63.6%) students knew that people cannot acquire HBV by holding hands with an infected person, and 168 (76.4%) answered correctly that HBV is more infectious than HIV/AIDS, while 161 (73.2%) knew that asymptomatic people can pass on HBV to others (Table 2).

### 3.3. Knowledge on Signs and Symptoms and Prevention of Hepatitis B

More than half 134 (60.6%) of the respondents knew that HBV-infected persons are asymptomatic at the acute phase. Regarding disease presentation, 127 (57.7%) participants were aware of jaundice as a sign of Hepatitis B infection and 133 (60.5%) knew that HBV can affect other organs order than the liver. Majority 189 (85.9%) of them knew that acute illness due to HBV causes liver inflammation. Concerning treatment and prevention, 154 (70.0%) said HBV infection can be treated and 214 (97.3%) knew that HBV is preventable by vaccination (Table 3).

### 3.4. Hepatitis B Testing and Vaccination History

About half 114 (50.4%) of the students studied had ever been tested for HBV infection. Of these, 44 (38.6%) got tested less than a year ago, while 70 (61.4%) got tested more than a year prior to this study. Among the students who were never tested for Hepatitis B infection, 82 (36.3%) had no reason for not getting tested, 19 (8.4%) said they could not afford to pay for the test, and 6 (2.7%) did not know where to have the test done. Other reasons given were fear of positive result 4 (1.7%) and did not have time 3 (1.3%). With regard to vaccination, 69 (30.5%) had received 3 completed dose, while 31 (13.7) received incomplete dose (1-2 doses). The rest 126 (55.6%) were never vaccinated against HBV. Regarding the reasons for not been vaccinated, majority 38 (16.8%) of them could not afford to pay for the vaccination, 37 (16.4%) did not have any reason, and 21(9.3%) said they did not receive the vaccine because they were not sick. Other reasons given included did not know where to go for vaccine, did not have time, and did not feel like vaccinating. Two (0.9%) did not receive the vaccine because they were HBV carriers (Table 4).

### 3.5. Factors Associated with Hepatitis B Testing

In the bivariate analysis, females were 1.26 times more likely to get tested for HBV compared to males, but this did not reach statistical significance. The odds of students who were 30 years and above getting tested was 3.27 times higher compared to students who were less than 20 years (p=0.0497). Students in their third year of study had 3.68 higher odds of being tested compared to their colleagues in their first year of study (p<0.001). Never married students were 42% less likely to get tested compared to those who were married/cohabiting (p=0.028). The likelihood of regular students to get tested for HBV infection was 0.39 times less compared to top-up students (p =0.0033). Students with good knowledge about HBV had 7.14 higher odds of been tested for the infection compared to their colleagues with poor knowledge (p=0.004).

After adjusting for confounding effect of the variables in the multivariate analysis, knowledge level had significant association with HBV testing. Students who had moderate knowledge (OR: 4.76; 95% CI: 1.35, 16.82; p=0.015) and those with good knowledge (OR: 5.41; 95% CI: 1.31, 22.36; p=0.020) were more likely to get tested for HBV compare to their colleagues with poor knowledge. In addition, students who were in their second year of study had 3.13 higher odds of been tested for HBV compared with those in their first year (Table 5).

### 3.6. Factors Associated with Receiving Full Dose of Hepatitis B Vaccine

In the bivariate analysis, females were 2.18 times more likely to receive complete dose of HBVc compared to males (95% CI: 1.13, 4.20; p=0.027). Never married students had 1.78 higher odds of being completely vaccinated compared to those who were married, though not statistically significant. Regular students were about 18% less likely to get fully vaccinated compared to top-up students (95% CI: 0.04, 0.75; p=0.0015). In the multivariate model, being a female showed a significant positive association with complete HBV vaccination (OR: 1.85; 95% CI: 1.04, 3.29; p=0.037) compared with males. Being a regular student (OR: 0.37; 95% CI: 0.19, 0.70; p=0.002) was a predictor for receiving partial or no HBV vaccination (Table 6).

## 4. Discussion

### 4.1. Knowledge of Hepatitis B among Undergraduate Students

In this study, we described the knowledge regarding HBV infection, its mode of transmission, signs and symptoms and prevention among Public Health undergraduate students of University of Health and Allied Sciences, Ghana. We also studied the students' HBV testing and vaccination history. It was observed that generally, the majority of students had moderate knowledge regarding HBV infection. Knowledge regarding the mode of transmission among participants is unsatisfactory. Having unprotected sex has been known to be the commonest route of transmission of HBV among adult population [2], however in this study, one-third of respondents disagree to this fact. Similarly, more than 4 in 10 of the respondents wrongly asserted that HBV is transmitted via the faeco-oral route and genes, while nearly half of them said the virus can infect a healthy individual through the air. This clearly indicates that there exist knowledge gap regarding the mode of transmission of the infection. Previous African studies also found knowledge of HBV among medical students and healthcare workers to be unsatisfactory. In northwest Ethiopia, for instance, 21% of medical students wrongly responded that HBV can be transmitted through the faeco-oral route, while nearly a quarter of them said the infection cannot be transmitted through unprotected sex [24]. In a similar study among Nigerian medical students, Desmond Aroke et al. reported that about 1 in 10 students did not know that HBV can be transmitted via needle stick [22]. Additionally, about 23% of public safety workers did not know the route of transmission of HBV and only 2% identified blood and other bodily fluids as a means through which the virus can be transmitted [25].

This is a worrying trend since HBV infection can pose a great occupational hazard to health workers due to their contact with infected individuals knowingly or unknowingly. Their unawareness regarding the modes of transmission of the infection may expose them to the infection and this could partly explain why HBV is still endemic in this part of the world. Ofori-Asenso and Agyeman in their systematic review estimated HBV prevalence of 12.3% among the general population and attributed this high prevalence to lack of adequate information and understanding of the transmission dynamics of the virus [6]. Among healthy blood donors in the Volta region, Osei et al. observed a seroprevalence of 7.5% and classified it as high-intermediate endemicity [7]. This is a clear indication that HBV infection remains a public health concern in Ghana, hence, the need to strengthen control and preventive measures to reduce the spread of the infection among HCWs and the general population.

In this study, almost half of the respondents answered incorrectly that jaundice is not a sign of HBV infection, and more than one-thirds of them were of the view that persons with HBV infection do not remain asymptomatic during the acute phase. These misconceptions regarding HBV disease presentation can influence health seeking behaviour of people with the infection. It has been established that readiness to seek medical care could be potentiated by factors, particularly cues to instigate action such as awareness of disease presentation [26]. Osei et al. reported that Tuberculosis patients without previous knowledge regarding the signs and symptoms of the disease were 5 times more likely to delay seeking medical diagnosis [27]. Expectedly however, Gedefew et al. found relatively high proportion of HCWs being aware that HBV-infected persons may be asymptomatic for a long time [24].

Awareness regarding vaccine as the main preventive measure against HBV infection is considerably high in this study and reached almost universal and is expected to positively influence the students' vaccination attitudes. This result is consistent with other previous studies in Cameroon [22] and Syria [19]. On the other hand, the respondents' incorrect assertion that HBV infection can be cured is a worrying misconception. Similar results were reported in previous study in Kumasi, Ghana [28].

### 4.2. Testing and Vaccination History among Students

Testing and diagnosis of HBV infection is the gateway for access to both prevention and treatment services and is an essential component of an effective response to the hepatitis epidemic. Testing provides an opportunity to link people to interventions to reduce transmission, through counselling on risk behaviours and hence the WHO recommends that all adults have routine access to and be offered HBV testing in the general population in settings with ≥2% or ≥5% sero-prevalence of Hepatitis B surface antigen (HBsAg) [1]. However, this current study observed unsatisfactorily low HBV testing rate among the students, results consistent with what was found among Saudi Arabia medical students [18] and among the USA population [20] but lower than what was reported among hospital workers in Nigeria [21]. In Syria, only 16% of medical students knew their HBV status [19]. This is a matter of concern since the prerequisite for HBV vaccination is for one to know his or her status. Hepatitis B testing and vaccination in Ghana outside the Expanded Program on Immunization (EPI) are not covered under the National Health Insurance scheme and hence one has to pay more than a dollar before getting tested. In addition, screenings for HBV are mainly prescribed at hospitals for patients suspected to be reactive to Hepatitis B and blood donors [6]. These could be attributed to the low testing rate among the students observed in this study. Though majority of the students claimed they had no reason for not testing for the infection, a significant proportion (17%) of them said they did not have money to go for the screening. For a country like Ghana, which is classified as a high burden for Hepatitis B to win the battle against the infection, it is important to consider making screening for the infection free of charge for everyone to have access to it irrespective of social class.

HBV vaccination has been recommended by the WHO as primary prevention strategy for the infection among healthcare workers [14]. In this present study, less than one-third of respondents had received 3 complete doses of HBV vaccine as at the time of the study. More than half of them were never vaccinated. This result is similar to what other studies reported [29, 30]. Because of their direct contact with patients or infective materials, HCWs including students are at considerably greater risk of HBV infection than the general population [31, 32] and hence need to be protected against the infection. The cost of vaccination may play a role in the low vaccination rate among the students. In this study, 3 in 10 students were not vaccinated because they could not afford to pay for it. In Ghana, one has to pay more than $4.0 to receive a single dose of HBVc. Poor and incomplete HBVc uptake among health workers and medical students have been reported by many studies [19–22, 25, 29, 32] and the cost of vaccination were cited by most of these studies as the main reason for low uptake.

### 4.3. Factors Associated with Hepatitis B Testing and Vaccination

Knowledge and level of study were identified in this study as predictors for HBV testing. Students with appreciable level of knowledge about the infection were over 4 times more likely to get tested than those with poor knowledge. This could be because students with good knowledge about Hepatitis B may be much informed on the health threats of the disease and the necessity to get tested to determine their status. The likelihood of HBV test increases with increasing number of years spent in school. This could be as a result of exposure to information about the disease in school since level of education has been found to be increase health awareness and subsequently health seeking.

Sex and been a top-up student were the predictors of receiving full dose of HBVc. Female students are more likely to be fully vaccinated compared to their male counterparts. Thus, females engage more on health seeking behaviour regarding HBV prevention more than males. Males typically are reluctance to consult healthcare providers and the under usage of healthcare service by men have been reported [33]. Ochu et al. observed in their study among safely workers that females had 2.28 times increased chance of receiving full dose of HBVc compared to males [25].

Expectedly, students who are currently not in any employment (regular students) are 37% less likely to be vaccinated against HBV. The employed students (top-ups) are trained health professionals who work in various health facilities in the country and are currently pursuing further education to upgrade their knowledge and skills in their area of specialty, so one can postulate that these category of students may have easy access to the HBVc at their work places and their ability to also afford to pay for the vaccination may account for the difference in vaccination status observed. Unlike this study, a study conducted among adolescents and young adults in Brazil reported a significant association between age and HBV vaccination status [34]. Similar study among dentists in Brazil on the other hand observed that age group and marital status do not have a significant association with HBV vaccination [35].

One obvious limitation of this study is that testing and vaccination status were self-reported and could not be verified by records; hence, recall bias may have an influence on these variables. We however tried to minimize this during data cleaning stage by getting the students to clarify any inconsistent responses.

## 5. Conclusion

The current study highlights the existence of significant knowledge gap regarding HBV transmission dynamics among the students. Additionally, the low uptake of HBV testing and incomplete or no vaccination among public health students may put them at risk of acquiring the infection. Good knowledge and higher level of study predict HBV testing, while female sex and been a top-up student were positively associated with receiving completed dose of HBVc. The University should therefore consider free screening and vaccination for students in order to protect this vulnerable group from exposure to the virus. Educational programs aimed at improving awareness about the infection are also required.

## Figures and Tables

**Table 1:** Characteristics of study participants.

Characteristics	Frequency	Percentage (%)
*Sex*		
Male	153	67.7
Female	73	32.3
*Age (in years)*		
<20	30	13.3
20 - 29	160	70.8
30+	36	15.9
*Level of study*		
1st year	57	25.2
2nd year	66	29.2
3rd year	59	26.1
4th year	44	19.5
*Program of study*		
Disease control	96	42.5
Health promotion	18	8.0
Public health general	4	1.8
Environmental health	11	4.9
Health information	40	17.7
Public Health Nutrition	57	25.2
*Religious affiliation*		
Christian	214	94.7
Muslim	8	3.5
Traditional	4	1.8
*Marital status*		
Married/co-habiting	31	13.7
Never married	195	86.3
*Type of student*		
Top-up	52	23.0
Regular	174	77.0
*Permanent residential status*		
Urban	181	80.1
Rural	45	19.9
*Source of funding for education*		
Self	43	19.0
Parents	163	72.1
Husband/wife	5	2.2
Other relative(s)	15	6.6

**Table 2:** Knowledge on causes and mode of transmission of HBV among students.

	Yes	No
Knowledge variable	n (%)	n (%)
Have heard of Hepatitis B infection	220 (97.4)	6 (2.6)
Hepatitis B is cause by a virus	207 (94.1)	13 (6.9)
Hepatitis B virus can be transmitted via unprotected sex	145 (65.9)	75 (34.1)
Hepatitis B virus can be transmitted via Blood transfusion	183 (83.2)	37 (16.8)
Hepatitis B virus can be transmitted via sharing towels with an infected person	138 (62.7)	82 (37.3)
Hepatitis B virus can be transmitted through the air	55 (25.0)	165 (75.0)
Hepatitis B virus be transmitted through the faeco-oral route	95 (43.2)	125 (56.7)
Hepatitis B virus can be transmitted through sharing sharps with an infected person	167 (75.9)	53 (25.1)
Hepatitis B infection is hereditary	103 (46.8)	117 (53.2)
Hepatitis B virus can be transmitted via holding hands with an infected person	80 (36.4)	140 (63.6)
Hepatitis B infection is more infectious than HIV/AIDS	168 (76.4)	52 (23.6)
Asymptomatic Hepatitis B patients can transmit the virus to others	161 (73.2)	59 (26.8)

**Table 3:** Knowledge on signs and symptoms and prevention of Hepatitis B.

	Yes	No
Knowledge variable	n (%)	n (%)
Infected people are asymptomatic at the acute phase	134 (60.9)	86 (39.1)
Jaundice is a sign of hepatitis B infection	127 (57.7)	93 (42.3)
Acute hepatitis B infection can result in liver inflammation	189 (85.9)	31 (14.1)
Hepatitis B can affect other organs other than the liver	133 (60.5)	87 (39.5)
Hepatitis B infection can be treated	154 (70.0)	66 (30.0)
Hepatitis B infection is preventable by vaccination	214 (97.3)	6 (2.7)

**Table 4:** Hepatitis B testing and vaccination among respondents.

Variable	Frequency	Percentage
*Tested for HBV*		
Ever tested	112	49.6
Never tested	114	50.4
*Last time got tested for HBV (N=112)*		
Less than a year ago	42	37.5
More than a year ago	70	62.5
*Reasons for not tested (N=114)*		
Do not have any reason	82	71.9
Do not have money	19	16.7
Do not know where to go	6	5.3
Do not have time	3	2.6
Fear of positive test result	4	3.5
*HBV vaccine up take*		
0 dose (not vaccinated)	126	55.8
1-2 doses (incomplete vaccination)	31	13.7
3 doses (complete vaccination)	69	30.5
*Reasons for not vaccinated (N=126)*		
Do not have any reason	37	29.4
Do not have money	38	30.2
Do not know where to go	19	15.1
Not sick	21	16.7
Others*∗*	11	8.7

Others*∗* include already a carrier: 2; no need: 3; do not have time: 3;

vaccine not available: 1; on medication: 1; and scared of needle: 1.

**Table 5:** Logistic regression analysis of factors associated with HBV testing.

Factors	OR (95% CI)	P-value	AOR (95% CI)	P-value
*Sex*		0.4218		
Male	1			
Female	1.26 (0.72-2.20)			
*Age group (years)*		0.0497		
<20	1		1	
20 – 29	1.52 (0.67-3.42)		1.04 (0.38, 2.83)	0.934
30+	3.27 (1.18-9.09)		1.18 (0.25, 5.55)	0.830
*Level of study*		0.0018		
1st Year	1		1	
2nd year	3.40 (1.61-7.20)		3.13 (1.30-7.52)	0.011
3rd year	3.68 (1.70-7.97)		2.30 (0.88, 6.05)	0.091
4th year	1.96 (0.86-4.46)		1.55 (0.58, 4.14)	0.377
*Programme of study*		0.6692		
Disease control	1		1	
Health promotion	1.00 (0.37-2.74)			
Mental Health	3.00 (0.30-29.87)			
Environmental Health	0.57 (0.16-2.08)			
Health information	0.74 (0.35-1.55)			
Nutrition	1.19 (0.62-2.30)			
*Religion*		0.2099		
Christian	1			
Muslim	0.33 (0.07-1.69)			
Traditionalist	3.00 (0.31-29.30)			
*Marital status*		0.0278		
Married/co-habiting	1		1	
Never married	0.42 (0.19-0.93)		0.94 (0.24, 3.60)	0.924
*Type of student*		0.0033		
Top-up	1		1	
Regular	0.39 (0.20-0.74)		0.80 (0.23, 2.82)	0.729
*Permanent residential status*		0.9201		
Urban	1			
Rural	0.97 (0.50-1.86)			
*Knowledge level*		0.0044		
Poor	1		1	
Moderate	3.95 (1.27-12.32)		4.76 (1.35, 16.82)	0.015
Good	7.14 (1.99-25.59)		5.41 (1.31, 22.30)	0.020

**Table 6:** Logistic regression analysis of factors associated with complete HBV vaccination.

Factors	OR (95% CI)	P-value	AOR (95% CI)	P-value
*Sex*		0.0277		
Male	1		1	
Female	2.18 (1.13, 4.20)		1.85 (1.04, 3.29)	0.037
*Age group (in years)*		0.1775		
<20	1			
20 – 30	0.85 (0.32, 2.30)			
31+	1.03 (0.22, 4.83)			
*Level of study*		0.8022		
1st year	1			
2nd year	1.48 (0.62, 3.56)			
3rd year	1.40 (0.49, 4.01)			
4th year	1.05 (0.38, 2.94)			
*Programme of study*		0.5394		
Disease control	1			
Health promotion	0.62 (0.19, 1.97)			
Mental health	0.49 (0.04, 5.29)			
Environmental health	0.22 (0.04, 1.15)			
Health information	1.71 (0.75, 3.92)			
Nutrition	0.67 (0.31, 1.41)			
*Religious affiliation*		0.5064		
Christian	1			
Muslim	0.31 (0.05, 1.83)			
Traditionalist	1.34 (0.14, 12.56)			
*Marital status*		0.2028		
Currently married/co-habiting	1			
Never married	1.78 (0.45, 6.95)			
*Type of student*		0.0015		
Top-up	1		1	
Regular	0.18 (0.04, 0.75)		0.37 (0.19, 0.70)	0.002
*Permanent residential status*		0.3264		
Rural	1			
Urban	0.66 (0.31, 1.40)			
*Knowledge level*		0.3264		
Good	1			
Moderate	2.42 (0.63, 9.24)			
Poor	1.82 (0.60, 5.47)			

## Data Availability

The data used to support the findings of this study are available from the corresponding author upon request.
